# Can a community health worker administered postnatal checklist increase health-seeking behaviors and knowledge?: evidence from a randomized trial with a private maternity facility in Kiambu County, Kenya

**DOI:** 10.1186/s12884-016-0914-z

**Published:** 2016-06-04

**Authors:** Margaret McConnell, Allison Ettenger, Claire Watt Rothschild, Faith Muigai, Jessica Cohen

**Affiliations:** Harvard T. H. Chan School of Public Health, Building 1, Room 1217, 665 Huntington Ave, Boston, MA 02115 USA; Jacaranda Health, P.O. Box 42844 – 00100, Nairobi, Kenya

**Keywords:** Postnatal care, Community health workers, Checklists, Mobile Health

## Abstract

**Background:**

Since the 2009 WHO and UNICEF recommendation that women receive home-based postnatal care within the first three days after birth, a growing number of low-income countries have explored integrating postnatal home visit interventions into their maternal and newborn health strategies. This randomized trial evaluates a pilot program in which community health workers (CHWs) visit or call new mothers three days after delivery in peri-urban Kiambu County, Kenya.

**Methods:**

Participants were individually randomized to one of three groups: 1) early postnatal care three days after delivery provided in-person with a CHW using a simple checklist, 2) care provided by phone with a CHW using the same checklist, or 3) a standard of care group. Surveys were conducted ten days and nine weeks postnatal to measure outcomes related to compliance with referrals, self-reported health problems for mother and baby, care-seeking behaviors, and postnatal knowledge and practices around the recognition of danger signs, feeding, nutrition, infant care and family planning.

**Results:**

The home visit administration of the checklist increased the likelihood that women recognized postnatal problems for themselves and their babies and increased the likelihood that they sought care to address those problems identified for the child. In both the home visit and mobile phone implementation of the checklist, actions taken for postnatal problems happened earlier, particularly for infants. Knowledge was found to be high across all groups, with limited evidence that the checklist impacted knowledge and postnatal practices around the recognition of danger signs, feeding, nutrition, infant care and family planning.

**Conclusion:**

We find evidence that CHW-administered postnatal checklists can lead to better recognition of postnatal problems and more timely care-seeking. Furthermore, our results suggest that CHWs can affordably deliver many of the benefits of postnatal checklists.

**Trial registration:**

ClinicalTrials.gov NCT02104635; registered April 2, 2014.

**Electronic supplementary material:**

The online version of this article (doi:10.1186/s12884-016-0914-z) contains supplementary material, which is available to authorized users.

## Background

Despite overall gains in child survival, slower progress has been made in reducing neonatal and maternal mortality in developing countries [1]. Globally, a critical gap in the continuum of maternal and newborn health has been identified during the early postnatal period [2]. Recommendations from the World Health Organization indicate crucial moments when contact with skilled health providers could be instrumental in identifying and responding to the needs and complications of both mother and baby after childbirth: the first few hours after birth, between three and seven days, and at 6 weeks [3]. Many countries are now struggling to develop logistically feasible models of postnatal care delivery that address the numerous challenges women face in returning to facilities in the short interval after birth.

Neonatal and maternal mortality rates continue to be high in Kenya, with an estimated 488 maternal deaths per 100,000 live births and 22 neonatal deaths per 1,000 live births based on the most recently available Demographic and Health Survey [4, 5]. Further, maternal and neonatal mortality rates are higher among the urban poor [6]. A major driver of poor maternal and neonatal outcomes is low utilization of postnatal care. Only 47 % of women in Kenya receive any postnatal care after delivery at all, and only 37 % of women receive postnatal care from a medical professional such as doctor, nurse or midwife [4]. Poor women with lower levels of education are even less likely to receive this essential care [5]. Decisions about whether to seek care, delays in recognizing illness and reaching facilities, and low-quality care at health service points have been documented in neighboring Uganda as barriers to access to this essential care for newborns and their mothers [7].

A growing body of evidence demonstrates that home visits by community health workers (CHWs) during pregnancy and the postnatal period reduced rates of low birth weight, stillbirths and neonatal mortality [8–11]. Such home visits have been found to increase key practices in the promotion of neonatal health including early initiation and exclusive breastfeeding, thermal care, hand hygiene, umbilical cord care and increased care-seeking behavior for sick infants [7, 12–15]. Additionally in some settings CHW-conducted home visits during the perinatal period demonstrated improvements in HIV-prevention strategies [8].

Despite the promise of early postnatal home visits, there is limited evidence on how to effectively implement postnatal care [16]. A Cochrane review of postnatal home visits examined 12 randomized trials both in high-resource and low-resource settings and found inconsistent results [17]. The review raises concerns that women who receive home visits may be less likely to seek care at facilities for their newborns if they feel they have already been sufficiently checked.

We conducted a randomized trial of a pilot program implemented three days after delivery in which a checklist was used by a community health worker to assess the health of the mother and newborn and targeted health education was offered. We compare the relative effectiveness of administering the checklist either in-person, as in more traditionally resource intensive CHW home visits, or by mobile phone, and compare both variations of the intervention to a standard of care group. The impact of the intervention with respect to identification of complications, care-seeking behaviors, health practices and knowledge was assessed.

## Methods

### Setting and study design

The intervention was implemented with Jacaranda Health, a private-sector social enterprise located in Kiambu County, Kenya providing high quality and low-cost maternal and newborn healthcare to low-income women in peri-urban areas of Nairobi. These regions are densely populated with characteristic challenges of poverty including poor access to water and sanitation, food insecurity and safety concerns [6, 18]. Total fertility rates for women in urban poor regions are slightly higher than that of Nairobi (3.1 compared to 2.8) yet still lower than the national average of 4.6 [4, 6].

At Jacaranda Health’s 10-inpatient bed hospital in a peri-urban setting just outside Nairobi, women were approached for recruitment and written informed consent after a normal delivery just prior to their discharge home. Women were eligible if they had a complication-free delivery, their newborn experienced no visible complications, were over 18 years old, provided two phone numbers where they could be reached, and resided within 20 km of the Jacaranda Health facility. These eligibility criteria reflect Jacaranda Health’s referral guidelines during the study period. During the study, Jacaranda Health had the capacity to conduct normal deliveries, provide basic emergency obstetric care and refer women who were at increased risk of a complication in their pregnancy to higher-level hospitals. Jacaranda's eligibility criteria were adapted from international guidelines including the World Health Organization and the U.K.’s National Institute for Clinical Excellence (NICE). The eligibility criteria excluded women with six or more past deliveries, advanced age (older than 35 years), abnormal values of ANC diagnostics (e.g. high blood pressure), history of medical complications, and history of obstetric complications.

The CHWs participating in our study were employed exclusively as Jacaranda staff and managed by a nurse midwife dedicated to outreach and community efforts. CHWs in our study were recruited from the larger area pool of CHWs who are recruited from barazas (local community meetings) and who receive foundational training in basic areas and are supervised by Community Health Extension Workers (CHEWs). The recruitment and training of these CHWs varies significantly in practice across Kenya [19].

The randomized trial took place between April 2014 and October 2014. Patients were individually randomized prior to enrollment using numeric patient identifiers assigned by Jacaranda Health. A unique identifier is given to each Jacaranda Health client seeking any service (including antenatal care, delivery, postnatal care, and child wellness care) during the client’s first visit to Jacaranda. Randomization was conducted by assigning each of these unique identifiers to one of the three central treatment groups with equal probability: a CHW home visit three days after delivery, a phone call from a CHW three days after delivery, or a standard of care group that received a customer service phone call about their experience.^1^. At the start of study recruitment, all pre-existing assigned Jacaranda identifiers were randomized. For clients who had attended antenatal care at Jacaranda, randomization was stratified using demographic and health variables collected in routine patient documentation. Identifiers were stratified by terciles of expected delivery date, distance from the client’s home to the facility, and primiparity. Random assignments were also given to all future Jacaranda IDs not yet in use; these assignments were made to each of the three treatment groups with equal probability. Random assignment of patient identifiers was done using a randomization sequence generated by the principal investigators with STATA 11, (StataCorp, College Station, TX).

### Intervention design

For women randomly assigned to the checklist groups, CHWs were trained to screen for maternal and newborn danger signs, to deliver targeted postnatal health education, and to refer mothers and their newborns to facility-based care if necessary using a checklist to guide them through the process that was available in English and Kiswahili (Additional file 1). Community health worker training was conducted by the research program manager and a designated nurse for the program. Prior to independent administration of the intervention, CHWs shadowed other Jacaranda Health nurses conducting postnatal health education counseling after delivery and observed a nurse-conducted home visit.

CHWs contacted women either in their home or by mobile phone three days after delivery. The checklist was developed using international guidelines and academic publications and included components shown in Table 1 [12, 20, 21]. Newborn assessment characteristics such as poor feeding, fever, and jaundice also reflect the 2014 WHO guidelines.^2^ If a CHW detected a sign or symptom of maternal or neonatal illness through the assessment checklist on Day 3, the mother or child were referred to the nearest facility for curative care.^3^ The protocol specified that all complications, except for cracked nipples in isolation, were to be referred. For all referrals, a nurse conducted a phone follow-up the following day to ensure the woman and her child received appropriate care. Drafts of the checklist were locally pre-tested to ensure accuracy and comprehension among the communities and CHWs. Managers conducted regular audits of referral and checklist documentation to ensure appropriate completion and adherence to protocols.

**Table 1:** Topics covered by day 3 postnatal checklist

Maternal	
*Screening criteria*	*Health education*
• Maternal infection (e.g. fever, mastitis) • Secondary postnatal hemorrhage • Postnatal Pre-eclampsia • Anemia	• Danger signs for postnatal care-seeking • Maternal nutrition • Postnatal family planning
Newborn	
*Screening criteria*	*Health Education*
• Insufficient breastfeeding/dehydration • Jaundice (palms, soles, and eyes) • Local infection (umbilical cord, eyes, and skin) • Breathing difficulties • Fever • Hypothermia	• Breastfeeding • Danger signs for newborn care-seeking • Umbilical cord care • Thermal care • Hand hygiene

CHWs completed a thorough four-day training to conduct screenings using the checklist and to counsel mothers and caregivers on essential postnatal health education. The training curriculum was adapted from international resources and modified to be relevant to specific programmatic contexts [20]. CHWs were evaluated through pre and post-test mechanisms to ensure thorough understanding and comprehension of essential information including: role-plays, observation of nurse-conducted visit and calls and, upon concluding the training, were signed-off by a clinical staff that their performance met quality standards.

### Data collection

Trained research assistants obtained written informed consent for participation in the study prior to discharge from the Jacaranda Health hospital in Ruiru. Recruitment and survey administration occurred on a rolling basis throughout the study period based on the date that women delivered at the Jacaranda Health hospitals.

Estimates of program impact were analyzed using administrative, programmatic, and survey data. Administrative data is routinely collected for all Jacaranda Health clients and uploaded into their medical records; these data include information on patient history and health services received, as well as demographic data. Electronic medical records data were available for all enrolled participants. The second data source was programmatic data collected by the CHW during administration of the checklist. The final source of data was follow-up participant surveys, administered by phone by trained research assistants at ten days and nine weeks after delivery.

All participants were contacted at each data collection time point, regardless of whether they were previously reached (including those who were never reached by either a home visit or phone call on the third day after giving birth). Inability to reach participants for both the day 3 interventions and the follow-up phone surveys was assessed based on the following protocols: For the day 3 interventions, participants assigned to receive a customer service call (standard of care arm) or phone administration of the checklist were contacted up to four times by phone on the scheduled day (using all phone contact information provided by the participant at enrollment). The participant was considered unreachable if none of the four calls were answered. For the home visit arm, the CHW contacted the participant by phone on the morning of her scheduled visit to confirm the location of the residence, and subsequently knocked on the door or otherwise introduced herself at the home. If the participant was not reachable by phone while the CHW was in the field that day or was not available at the home for the entire day, she was considered unable to be reached by the checklist treatment.

Regardless of whether the participants were reached on day 3, all were scheduled for follow-up mobile phone surveys at ten days and nine weeks after delivery. Attempts were made to contact participants for the day 10 follow-up survey beginning on day 10 after delivery, with daily contact attempts made for the subsequent seven days after the scheduled date. If the participant was unreachable or unable to complete the phone survey during this time, she was considered unreachable. Likewise, attempts were made to contact participants for the nine week survey beginning on the scheduled date, with daily contact attempts made for a subsequent 14 days.

### Outcomes

Postnatal maternal and newborn outcomes are captured at three time periods – three days, ten days, and nine weeks post-delivery. Day 3 outcomes were collected during administration of the checklist by the CHW and include maternal or newborn complications detected and referrals made for complications. These outcomes were collected in both treatment arms but not the standard of care arm. Day 10 and 9 week survey outcome measures include care-seeking behaviors for mother and newborns and both knowledge and practice of infant care, nutrition, feeding and recognition of danger signs. Table 2 provides definitions of all outcome measures.

**Table 2:** Definition of primary outcomes

*Outcome measure*	*Definition*
*Outcomes measured at 3 days postpartum*	
Maternal complication detected and referred	CHWs documented any observed or reported maternal complications detected through administration of the checklist; data collected for study participants assigned to CHW treatment by phone or home visit
Newborn complication detected and referred	CHWs documented any observed or reported complications detected through administration of the checklist; data collected for study participants assigned to CHW treatment by phone or home visit
*Outcomes reported at 10 days postpartum*	
Maternal referral acted upon	Binary variable indicating CHW referral was made (from programmatic data) and respondent reporting seeking facility-based care based on referral advice
Newborn referral made and acted upon	Binary variable indicating CHW referral was made (from programmatic data) and respondent reporting seeking facility-based care based on referral advice
Facility-based maternal postpartum care sought	Binary variable indicating whether respondent reported going to a health facility for her health since delivery
Days postpartum maternal care sought, among maternal care-seekers	Continuous variable indicating days after delivery maternal care was sought, for those who report seeking facility-based maternal care
Facility-based newborn postpartum care sought	Binary variable indicating whether respondent reported bringing her baby for a child wellness clinic visit
Days postpartum newborn care sought, among maternal care-seekers	Continuous variable indicating days after delivery newborn wellness care was sought, for those who report seeking facility-based newborn wellness care
Any maternal health problem reported	Binary variable indicating respondent reported yes to the question, “Are you having any problems with your health, related to your delivery, since you delivered?”
Maternal problem reported and action taken	Binary variable indicating respondent reported yes to the question, “Did you do anything as a response to this problem?”, administered to respondents who reported a newborn health problem.
Any newborn health problem reported	Binary variable indicating respondent reported yes to the question, “Is your baby having any problems with his/her health since you delivered?”
Newborn problem reported and action taken	Binary variable indicating respondent reported yes to the question, “Did you do anything as a response to this problem?”, administered to respondents who reported a newborn health problem.
Number of maternal danger signs named	Accurate identification of maternal danger signs defined as ability to name any of the following 11 predefined signs: fever/chills, foul-smelling vaginal discharge, convulsions/loss of consciousness, heavy vaginal bleeding (defined as soaking through a pad every hour), severe headaches, dizziness or faintness, visual disturbance (blurry vision or unusual difficulty seeing), increased cramping or abdominal pain, increased perineal pain, swelling, redness or discharge, difficulty passing urine, difficulty breathing, breast redness or hot to the touch, and pain or lump in the breast.
Can name 3 or more maternal danger signs	Binary variable coded for continuous outcome variable, indicating whether 3 or more maternal dangers signs were named
Number of newborn danger signs named	Accurate identification of infant danger signs defined as ability to name any of the following eight signs: fever, jaundice, poor feeding, lethargic/unresponsive, umbilical cord redness or discharge, convulsions, abnormal breathing (including panting, fast breathing, grunting, or nasal flaring, and pus from eyes.
Can name 3 or more newborn danger signs	Binary variable coded for continuous outcome variable, indicating whether 3 or more newborn dangers signs were named
Can name 2 or more hand washing best practices	Accurate identification of hand washing best practices defined as ability to name any of the following times that are particularly important for a caregiver to wash hands: after using the toilet, before touching/holding baby, after washing or touching nappies
Can name 2 or more cord care practices	Accurate identification of sources of cord care practices defined as ability to name any of the following best practices to prevent cord infection: keep cord clean and dry, do not apply anything to stump, keep cord outside nappy/diaper
Can name 3 or more newborn thermal care practices	Accurate identification of sources of dietary protein defined as ability to name any of the following best practices to ensure baby stays warm: keep room where newborn stays warm, dress newborn in several layers of clothes, bathe baby quickly in cold weather using warm water and dry and dress baby quickly, keep newborn’s head covered, practice skin to skin contact
Can name 3 or more sources of dietary protein	Accurate identification of sources of dietary protein defined as ability to name any of the following: beans, lentils, meat, eggs, chicken, fish, milk
Applied water or nothing to umbilical stump	Binary variable indicating nothing or water applied to umbilical stump. The checklist and data collection defined best practices in umbilical cord care according to Jacaranda Health’s clinical practice of dry cord care at the time of data collection. While Kenya introduced a change in its newborn care clinical guidelines from dry care to use of topical antiseptic in November 2013, the World Health Organization recommends both dry cord care and topical antiseptic as best practice [33, 34].
Appropriate newborn thermal care practiced	Binary variable indicating respondent mentions trying to keep baby warm when asked how she bathes her baby
Exclusive breastfeeding	Binary variable indicating only newborn consumed only breastmilk since birth
Breastfed 3 or more times in past 8 hours	Binary variable indicating respondent reports feeding baby 3 or more times in 8 hours prior to the survey
*Outcomes reported at 9 weeks postpartum*	
At least one dose of polio and pentavalent vaccines	Binary variable indicating that respondent reports infant has received at one or more doses of polio vaccine and one or more doses of pentavalent vaccine when asked which vaccinations the infant has received since delivery
Use of family planning method	Binary variable indicating respondent reports current use of any family planning method
Exclusive breastfeeding	Binary variable indicating only newborn consumed only breastmilk since birth
Breastfed 3 or more times in past 8 hours	Binary variable indicating respondent reports feeding baby 3 or more times in 8 hours prior to the survey
*Index measures*	
Index of health knowledge at 10 days post-delivery	Summative index with a maximum of 6 points and a minimum of 0 points, where each point represents knowledge of the following 6 postnatal health topics: 1) ability to name 3 or more maternal danger signs; 2) ability to name 3 or more infant danger signs; 3) ability to name 2 or more hand washing best practices; 4) ability to name 2 or more; 5) ability to name 3 or more recognized newborn thermal care practices; 6) and ability to name 3 or more sources of maternal dietary protein
Index of health practices at 10 days and 9 weeks post-delivery	Summative index with a maximum of 8 points and a minimum of 0 points, where 4 points represent 4 key health practices reported at 10 day (exclusive breastfeeding, breastfed 3 or more times in the past 8 hours, appropriate newborn thermal care practices, and water or nothing applied to umbilical cord stump) and 4 points represent 4 key health practices reported at 9 weeks (exclusive breastfeeding, breastfed 3 or more times in the past 8 hours, use of postnatal contraception, infant has received at least one dose of polio and pentavalent vaccines).

In order to avoid concerns about multiple outcome testing, levels of postnatal health knowledge were assessed by constructing summative indices of the number of items that participants were able to name in response to knowledge questions as suggested by O’Brien [22] and Kling and Liebman (2004) [23]. Knowledge outcomes were also converted into binary variables, indicating whether the participant was able to name a specified number of knowledge items.

### Ethical approval trial registration

This study was approved by Institutional Review Boards at Harvard School of Public Health and the Ethical and Scientific Review Committee of African Medical Research Foundation (AMREF) in Nairobi, Kenya. The study design was registered at clinicaltrials.gov with identification number NCT02104635.

### Statistical power

Because of the small sample of this study, our study is powered to see only large changes in the primary care-seeking outcomes. With our original registered sample size of 109 individuals, we had 80 % power to detect a change of 30 percentage points in maternal care-seeking from a base of 8 % of women seeking care, using a two-sided test comparing any two treatment groups and a 5 % significance level threshold. Because child care seeking levels are so high, even with the standard of care (over 95 % of the sample seeks some care for their newborn prior to the 10 day survey), we were not powered on this outcome. Because of loss to follow-up our actual sample size for the survey conducted 10 days after delivery was 83. With our final sample, we have 80 % power to detect a change of 37 percentage points in maternal care-seeking compared to the standard of care, using a two-sided pairwise test and a 5 % significance level. Power calculations were conducted using STATA, version 12.1.

### Data analysis

We test for differences in outcomes across study arms on an intention-to-treat basis, with the treatment arms defined as participants’ randomized treatment assignment and the sample including all respondents where data is available (not just those reached by a day 3 intervention). We also provide Additional file 2: Tables A1-A3, which recreate all regression analyses restricted to the subset of participants who were successfully reached for the day 3 intervention.

Program impacts were estimated using logistic regression for binary outcomes and ordinary least squares (OLS) regression for continuous outcomes. OLS and logistic estimates of program impact are presented both in unadjusted and adjusted models. Outcomes of interest are regressed on separate binary variables for assignment to one of the treatment arms. Adjusted estimates include controls for female participant’s age, marital status, employment status in the past 12 months, attendance at one or more antenatal care visits at a Jacaranda Health facility, and a binary indicator for whether the participant was enrolled in a postnatal contraceptive subsidy program as part of a concurrent randomized controlled trial. All analyses were performed using Stata software, version 12.1 (StataCorp, College Station, TX).

**Fig. 1:**
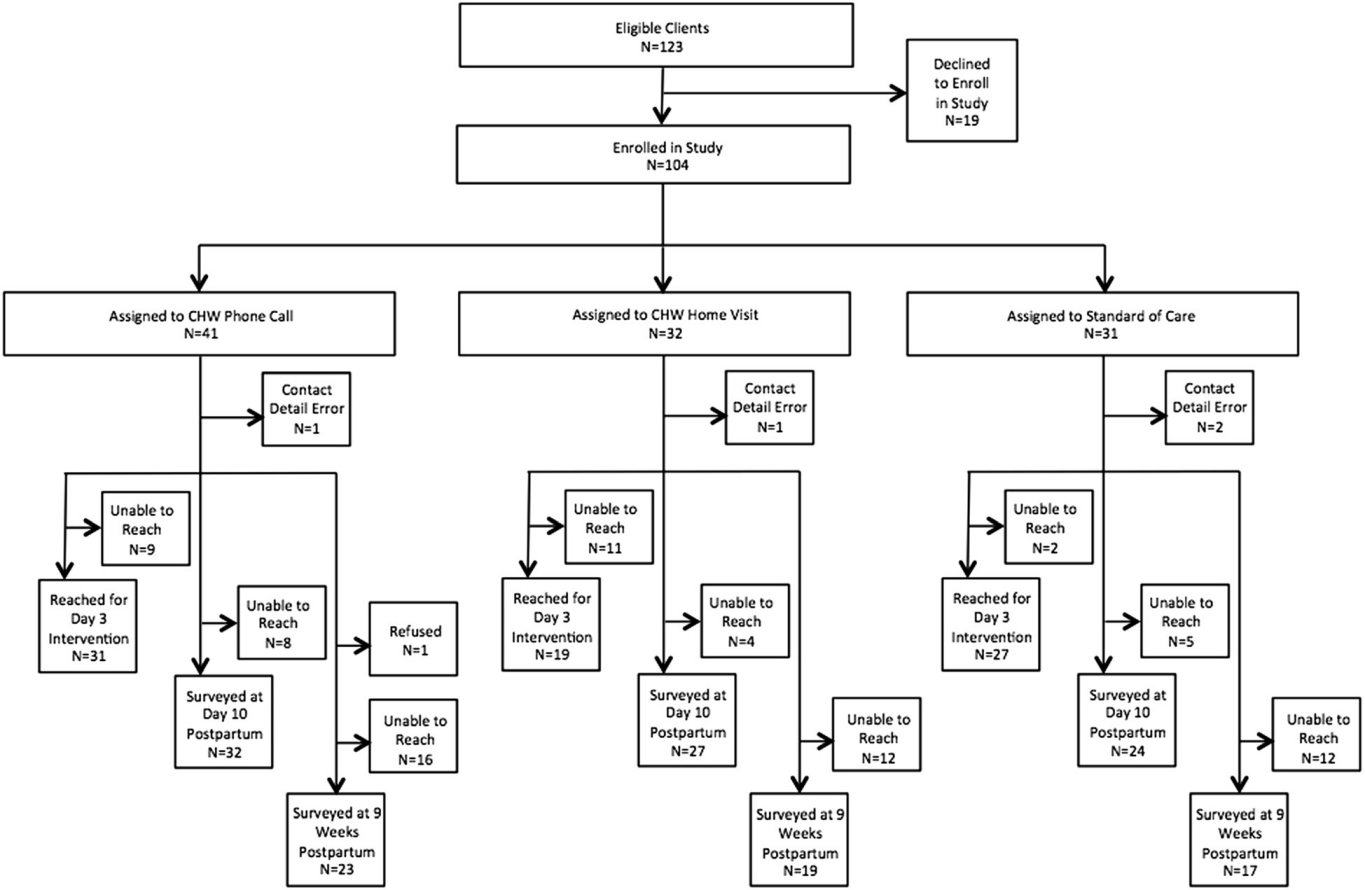
Randomized trial study design and participant flow

Participant flow through enrollment, administration of the day 3 interventions, and follow-up surveying are presented in Fig. 1. Programmatic data were collected for the 77 women who were successfully reached at day 3 by the assigned intervention. The rate of reaching individuals at day 3 for the intervention was higher in the checklist call group (76 %) compared to the checklist home visit group (59 %). This difference may be due to difficulties finding participants either because they are traveling or because their households were difficult to locate. Participant survey data were collected for 83 participants at ten days post-delivery (24 in the standard of care arm, 32 in the phone-administered checklist arm, and 27 in the home visit-administered checklist arm). Participant surveys were collected for 59 participants at nine weeks after delivery (17 in the standard of care arm, 23 in the phone arm, and 19 in the home visit arm). Four participants were unable to be contacted at any data collection point after enrollment, due to errors in their contact details.

## Results

Demographic and household characteristics of the sample included as controls in adjusted models are presented by treatment arm in Table 3. Participants are on average 26–27 years old, with less than 1 % of the total sample older than 35. The majority of participants are married, have received at least some secondary schooling, and have had previous pregnancies. Most of the differences in characteristics across arms are small and not statistically significant, except for a somewhat lower rate of marriage in the call arm (85 % (35/41) vs. 97 % (30/31) and 97 % (30/31) in the standard of care and home visit arms, respectively) and a lower employment rate in the visit arm (41 % (11/27) vs. 66 % (21/32) to 70 % (16/23) in the other arms). While the difference in employment status in the visit arm is substantial, it does not seem to reflect overall imbalance in socioeconomic status across arms, as households in the visit arm are not significantly less educated or less likely to have improved toilets or water supplies. A subsample of our participants was concurrently enrolled in a study that provided a voucher for free postpartum family planning. Since assignment to the voucher arm was higher in the CHW call group arm we include this voucher assignment as a control in all adjusted models.

**Table 3:** Descriptive statistics of participant demographics and primary outcomes

Panel A. Participant demographic and household characteristics									
		Standard of care		CHW call		CHW visit		*p*-values	
		*n*	% / mean	*n*	% / mean	*n*	% / mean	CHW call vs. Standard of care	CHW visit vs. Standard of care
Women									
Age		31	26.7	40	25.6	31	25.9	0.23	0.38
Education									
	Primary	4	14 %	8	21 %	4	15 %	0.59	0.49
	Secondary	15	52 %	18	47 %	16	59 %		
	Post-Secondary	10	35 %	12	32 %	7	26 %		
	n	19		38		27			
Marital Status									
	Married	30	97 %	35	85 %	30	97 %	0.08	>0.99
	Single	1	3 %	6	15 %	1	3 %		
	n	31		41		31			
Parity									
	Primiparous	9	29 %	8	20 %	9	30 %	0.36	0.94
	Multiparous	22	71 %	33	81 %	21	70 %		
	n	31		41		31			
Employed in Past 12 Months		16	70 %	21	66 %	11	41 %	0.76	0.04
	n	23		32		27			
Assigned to postnatal contraception subsidy treatment through concurrent randomized trial		6	19 %	14	34 %	7	22 %	0.16	0.81
	n	31		41		32			
Husband									
Husband Education									
	Primary	0	0 %	4	14 %	2	8 %	0.12	0.11
	Secondary	15	65 %	13	45 %	10	39 %		
	Post-Secondary	8	35 %	12	41 %	14	54 %		
	n	23		29		26			
Household									
Improved toilet facility		24	92 %	34	97 %	24	86 %	0.43	0.45
	n	26		35		28			
Toilet shared among more than one house		9	39 %	15	47 %	10	39 %	0.58	0.96
	n	23		32		26			
Improved drinking water source		18	90 %	27	96 %	23	92 %	0.41	0.82
	n	20		28		25			
Panel B. Primary outcomes									
		Standard of Care		CHW call		CHW visit		p-values	
		n	% / mean	n	% / mean	n	% / mean	CHW call vs. Standard of care	CHW visit vs. Standard of care
Complications detected and referred, collected through programmatic CHW data									
Maternal or newborn complications detected and referred by CHW				4	10 %	3	9 %		
	n			41		32			
Postpartum care-seeking practices for mother and newborn, reported during day 10 survey									
Referrals made and acted upon				1	4 %	2	11 %		
	n			23		18			
Facility-based maternal postpartum care sought		2	8 %	4	13 %	6	22 %	0.62	0.17
	n	24		32		27			
Days postpartum maternal care sought, among maternal care-seekers		2	8.5	4	7.5	6	5.8	0.57	0.09
Facility-based newborn postpartum care sought		23	96 %	30	94 %	26	96 %	0.73	0.93
	n	24		32		27			
Days postpartum newborn care sought, among newborn care-seekers		23	5.9	30	3.8	26	4.1	0.02	0.04
Postnatal health problems and responses at day 10 post-delivery									
Any maternal health problem reported		3	13 %	2	6 %	9	33 %	0.48	0.08
	n	24		32		27			
Maternal problem reported and action taken		2	8 %	2	6 %	3	11 %	0.77	0.74
	n	24		32		27			
Any newborn health problem reported		5	21 %	8	25 %	13	48 %	0.72	0.04
	n	24		32		27			
Newborn problem reported and action taken		3	13 %	5	16 %	8	30 %	0.74	0.14
	n	24		32		27			
Self-reported knowledge of postnatal danger signs and health practices at day 10									
Number of maternal danger signs named		24	3.8	32	4.1	27	4.3	0.46	0.23
Can name 3 or more maternal danger signs		19	79 %	26	81 %	23	85 %	0.85	0.56
	n	24		32		27			
Number of newborn danger signs named		24	3.3	32	3.7	27	3.6	0.41	0.47
Can name 3 or more newborn danger signs		19	79 %	26	81 %	23	85 %	0.36	0.13
	n	24		32		27			
Can name 2 or more hand washing best practices		10	42 %	19	59 %	10	37 %	0.20	0.74
	n	24		32		27			
Can name 2 or more cord care practices		7	29 %	13	41 %	5	19 %	0.38	0.39
	n	24		32		27			
Can name 3 or more newborn thermal care practices		7	29 %	11	34 %	8	30 %	0.68	0.97
	n	24		32		27			
Can name 3 or more sources of dietary protein		7	29 %	12	38 %	11	41 %	0.52	0.40
	n	24		32		27			
Self-reported postnatal health practices at day 10									
Applied water or nothing to umbilical stump		17	71 %	26	81 %	23	85 %	0.38	0.23
	n	24		32		27			
Appropriate newborn thermal care practiced		17	71 %	26	81 %	23	85 %	0.84	0.93
	n	24		32		27			
Exclusive breastfeeding		24	100 %	32	100 %	27	100 %	--	--
	n	24		32		27			
Breastfed 3 or more times in past 8 hours		23	96 %	27	84 %	25	93 %	0.14	0.63
	n	24		32		27			
Self-reported postnatal health practices at 9 weeks post-delivery									
At least 1 dose of polio and pentavalent vaccines		15	88 %	22	96 %	18	95 %	0.43	0.50
	n	17		23		19			
Use of family planning method		5	29 %	14	61 %	8	42 %	0.05	0.44
	n	17		23		19			
Exclusive breastfeeding		17	100 %	21	91 %	19	100 %	0.16	--
	n	17		23		19			
Breastfed 3 or more times in past 8 hours		15	88 %	22	96 %	18	95 %	0.43	0.50
	n	17		23		19			
Indices of postnatal health knowledge and practices at 10 days and 9 weeks post-delivery									
Index of health knowledge at 10 days post-delivery		24	3.17	32	3.56	27	3.15	0.37	0.96
Index of health practices at 10 days and 9 weeks post-delivery		15	6.6	20	7.1	17	7.2	0.16	0.06

### Postnatal complication detection and referral at 3 days after delivery

At the day 3 visit, CHWs documented any observed or reported maternal and newborn complications detected through administration of the checklist. Complications were detected and referrals made for 10 % (4/41) and 9 % (3/32) of the phone call and home visit arms, respectively, with no significant difference in referral percentage by arm (unadjusted 95 % CI: −0.14–0.14; p = 0.957). All complications detected related to newborn danger signs, with no maternal complications detected in any arm. As reported by clients at day 10 survey follow-up, 4 % (1/23) and 11 % (2/18) of participants both had a referral and acted upon it in the phone call and home visit arms, respectively. No difference was found in referrals acted upon in the home visit relative to the phone call arms (unadjusted 95 % CI: −0.11–0.25; *p* = 0.445).

### Care-seeking practices at 10 days after delivery

Table 4 presents participant-reported health-seeking practices at the ten-day postnatal follow-up survey. Panel A presents maternal-focused care sought for any reason at a health facility, including routine postnatal care. Relative to 8 % (2/24) of women in the standard of care arm who sought maternal care, participants in the phone call treatment arm were 1.6 times more likely to have sought maternal care by day 10 (unadjusted 95 % CI: 0.26–9.49; *p* = 0.622), while participants in the home visit arm were three times as likely (unadjusted 95 % CI: 0.56-17.5; *p* = 0.192). Differences between the standard of care arm and each treatment arm are not statistically significant in the crude model; facility-based care seeking is higher in the home visit arm relative to the standard of care arm in the adjusted model (adjusted 95 % CI: 0.77-71.5; *p* = 0.082), though the result is not quite significant at the 5 % level. Relative to maternal care seeking, facility-based infant-related care seeking was very high across arms (Panel B): 96 % (23/24) of standard of care arm participants reported seeking infant-related care (including immunizations and child wellness visits) by ten days after delivery, as compared to 94 % (30/32) and 96 % (26/27) of the phone and visit arms, respectively.

**Table 4:** Postnatal care-seeking practices for mother and newborn reported during day 10 survey

Panel A. Maternal postnatal care seeking				
	(1)	(2)	(3)	(4)
	Facility-based maternal postnatal care sought	Facility-based maternal postnatal care sought	Days postnatal maternal care sought, among maternal care-seekers	Days postnatal maternal care sought, among maternal care-seekers^‡^
	*Unadjusted*	*Adjusted*	*Unadjusted*	*Adjusted*
*Estimation Type*	*Logistic*	*Logistic*	*OLS*	*OLS*
CHW phone call	1.57	1.63	−1	−1.05
	(0.26 - 9.49)	(0.23 - 11.8)	(−4.52 - 2.52)	(−10.3 - 8.23)
CHW home visit	3.14	7.44*	−2.67*	−2.27
	(0.56 - 17.5)	(0.77 - 71.5)	(−5.66 - 0.33)	(−14.2 - 9.65)
Standard of care arm mean	0.08	0.08	8.5	8.5
p-value for test of call = visit	0.330	0.105	0.417	0.769
p-value for test of joint significance of call and visit	0.366	0.161	0.173	0.833
Controls?	N	Y	N	Y
R-squared			0.159	0.438
Observations	83	79	12	10
Panel B. Newborn postnatal care seeking				
	(1)	(2)	(3)	(4)
	Facility-based newborn postnatal care sought^‡‡^	Facility-based newborn postnatal care sought^‡^	Days postnatal newborn care sought, among newborn care-seekers	Days postnatal newborn care sought, among newborn care-seekers
	*Unadjusted*	*Adjusted*	*Unadjusted*	*Adjusted*
*Estimation Type*	*Logistic*	*Logistic*	*OLS*	*OLS*
CHW phone call	0.65	0.47	−2.04**	−2.27***
	(0.055 - 7.76)	(0.054 - 4.10)	(−3.65 - -0.42)	(−3.89 - -0.64)
CHW home visit	1.13	0.51	−1.79**	−1.78*
	(0.066 - 19.4)	(0.022 - 11.8)	(−3.45 - -0.14)	(−3.69 - 0.13)
Mean of standard of care arm	0.96	0.96	5.86	5.86
p-value for test of call = visit	0.663	0.942	0.714	0.537
p-value for test of joint significance of call and visit	0.891	0.791	0.041	0.025
Controls?	N	Y	N	Y
R-squared			0.098	0.195
Observations	83	40	79	75

*** *p* < 0.01, ** *p* < 0.05, * *p* < 0.1

^‡^ The binary covariate for receipt of a postnatal contraception subsidy is dropped due to collinearity.

^‡‡^ The reduced number of observations in Panel B (columns 1 and 2) is due to the low variation in the outcomes across treatment arms, with 94.3 % of the total sample reporting having sought newborn care by the day 10 survey. In model (1), the coefficient on the CHW phone call intervention cannot be estimated due to this lack of variation in the outcome relative to the standard of care arm. Likewise, no adjusted model is presented in column (2) due to the reduced number of observations when covariates are included in the model. Notes: Odds ratios generated via logistic regression (models 1, 2) are presented with 95 % confidence intervals constructed with robust standard errors. Maternal care seeking (panel A, columns 1 and 2) is defined as a binary variable, taking on the value of 1 if the woman reports seeking facility-based maternal-related care. Infant care seeking (panel B, columns 1 and 2) is defined as a binary variable, taking on the value of 1 if the woman reports attending a child wellness visit. OLS coefficients are presented for models 3 and 4. Days postnatal maternal care sought (panel A, columns 3 and 4) is defined as a continuous variable of the number of days that maternal care was sought after the date of delivery. Days postnatal infant care sought (panel B, columns 3 and 4) is likewise defined as a continuous variable of the number of days that care for the infant was sought after the date of delivery. For all adjusted regressions (columns 2 and 4), individual level covariates include the female participant’s age (coded as an ordinal variable with values 18–25, 26–30, 31–35, or 36–40 years old), marital status (defined as a binary single or married), a binary variable indicating whether the female respondent was employed at any time in the past 12 months, a binary variable indicating whether the participant was enrolled in a concurrent randomized trial and received a voucher (cost subsidy) for postnatal family planning services, and a binary variable indicating whether the participant attended one or more antenatal care visits at a Jacaranda Health facility

We find evidence of program impacts on the timing of care-seeking for both mother and infant. Among participants who reported seeking facility-based maternal care, participants assigned to the home visit arm sought care on average 2.7 days sooner than the standard of care arm (unadjusted 95 % CI: −5.66–0.33; *p* = 0.075), though this result is not quite statistically significant at the 5 % level. Statistically significant differences are found in the timing of infant-related care seeking: among participants who sought facility-based infant-related care, assignment to the phone call and home visit resulted in infant care-seeking 2.0 (*p* = 0.014) and 1.8 days (*p* = 0.034) earlier than the standard of care.

In Table 5, we present self-reported maternal (Panel A) and newborn (Panel B) health problems and responses taken. 13 % (3/24) of women assigned to the standard of care arm reported experiencing a maternal health problem, and 8 % (2/24) both experiencing a problem and reporting taking action to address this health problem. Assignment to the home visit is estimated to increase the likelihood of reporting a maternal problem by 3.5 times (unadjusted 95 % CI: 0.81-15.1; *p* = 0.092) to 5.6 times (adjusted 95 % CI: 0.88-35.4; *p* = 0.068) relative to the standard of care group, depending on model specification, though these effects do not meet a standard of 5 % significance level. The odds of reporting a maternal health problem in the call arm were not significantly different from the standard of care group and were significantly higher in the home visit arm than in the call arm (unadjusted 95 % CI: 1.43–39.20; *p* = .017). While the odds of reporting a maternal problem and taking action were higher in the home visit arm than in the standard of care arm, these differences were not statistically significant.

**Table 5:** Postnatal health problems and responses as reported during day 10 survey

Panel A. Maternal health problem reporting and responses				
	(1)	(2)	(3)	(4)
	Any maternal health problem reported	Any maternal health problem reported	Maternal problem reported and action taken	Maternal problem reported and action taken
	*Unadjusted*	*Adjusted*	*Unadjusted*	*Adjusted*
*Estimation Type*	*Logistic*	*Logistic*	*Logistic*	*Logistic*
CHW Call	0.47	0.23	0.73	0.50
	(0.071 - 3.08)	(0.021 - 2.58)	(0.095 - 5.69)	(0.047 - 5.39)
CHW Home Visit	3.50*	5.58*	1.38	1.69
	(0.81 - 15.1)	(0.88 - 35.4)	(0.21 - 9.12)	(0.18 - 15.5)
Mean of standard of care arm	0.13	0.13	0.83	0.83
p-value for test of call = visit	0.017	0.004	0.512	0.371
p-value for test of joint significance of call and visit	0.032	0.009	0.805	0.670
Controls?	N	Y	N	Y
Observations	83	79	83	53
Panel B. Newborn health problem reporting and responses				
	(1)	(2)	(3)	(4)
	Any newborn health problem reported	Any newborn health problem reported	Newborn problem reported and action taken	Newborn problem reported and action taken
	*Unadjusted*	*Adjusted*	*Unadjusted*	*Adjusted*
*Estimation Type*	*Logistic*	*Logistic*	*Logistic*	*Logistic*
CHW Call	1.27	1.21	1.30	0.95
	(0.35 - 4.54)	(0.33 - 4.46)	(0.28 - 6.11)	(0.20 - 4.57)
CHW Home Visit	3.53**	3.78*	2.95	3.94*
	(1.01 - 12.3)	(0.95 - 15.1)	(0.68 - 12.9)	(0.80 - 19.4)
Mean of standard of care arm	0.21	0.21	0.13	0.13
*p*-value for test of call = visit	0.070	0.087	0.205	0.053
*p*-value for test of joint significance of call and visit	0.078	0.120	0.260	0.104
Controls?	N	Y	N	Y
Observations	83	79	83	79

*** *p* < 0.01, ** *p* < 0.05, * *p* < 0.1

Notes: Odds ratios generated via logistic regression (models 1–4) are presented with 95 % confidence intervals constructed with robust standard errors. “Any maternal problem reported” is a binary variable indicating whether or not the participant reported any maternal-related health problems at 10 days postnatal. “Maternal problem reported and action taken” is a binary variable taking the value 1 if the respondent reported both any maternal health problem or concern and taking any action to address the problem, including calling a health facility or pharmacy, visiting a facility or pharmacy, or another action. “Any infant problem reported” is a binary variable indicating whether or not the participant reported any infant-related health problems at 10 days postnatal. “Newborn problem reported and action taken” is a binary variable taking the value 1 if the respondent reported both any newborn health problem or concern and taking any action to address the problem, including calling a health facility or pharmacy, visiting a facility or pharmacy, or another action. For all adjusted regressions (columns 2 and 4), individual level covariates include the female participant’s age (coded as an ordinal variable with values 18–25, 26–30, 31–35, or 36–40 years old), marital status (defined as a binary single or married), a binary variable indicating whether the female respondent was employed at any time in the past 12 months, a binary variable indicating whether the participant was enrolled in a concurrent randomized trial and received a voucher (cost subsidy) for postnatal family planning services, and a binary variable indicating whether the participant attended one or more antenatal care visits at a Jacaranda Health facility

We find that 21 % (5/24) of women assigned to the standard of care arm reported newborn health problems, with 13 % (3/24) reporting both noticing and responding to a newborn health problem. Women assigned to the home visit were 3.5 times (unadjusted 95 % CI: 1.01-12.3; *p* = 0.048) to 3.8 times (adjusted 95 % CI: 0.95-15.1; *p* = 0.060) more likely to report newborn health problems. Women in the home visit arm were 2.95 (unadjusted 95 % CI: 0.68-12.9; *p* = 0.151) more likely to both report and take action on a newborn problem than in the standard of care group in the unadjusted model, and 3.94 (adjusted 95 % CI: 0.80-19.4; *p* = 0.092) times more likely to do so in the adjusted model, although estimates are not statistically significant at the 5 % level.

### Postnatal knowledge at 10 days after delivery

**Fig. 2:**
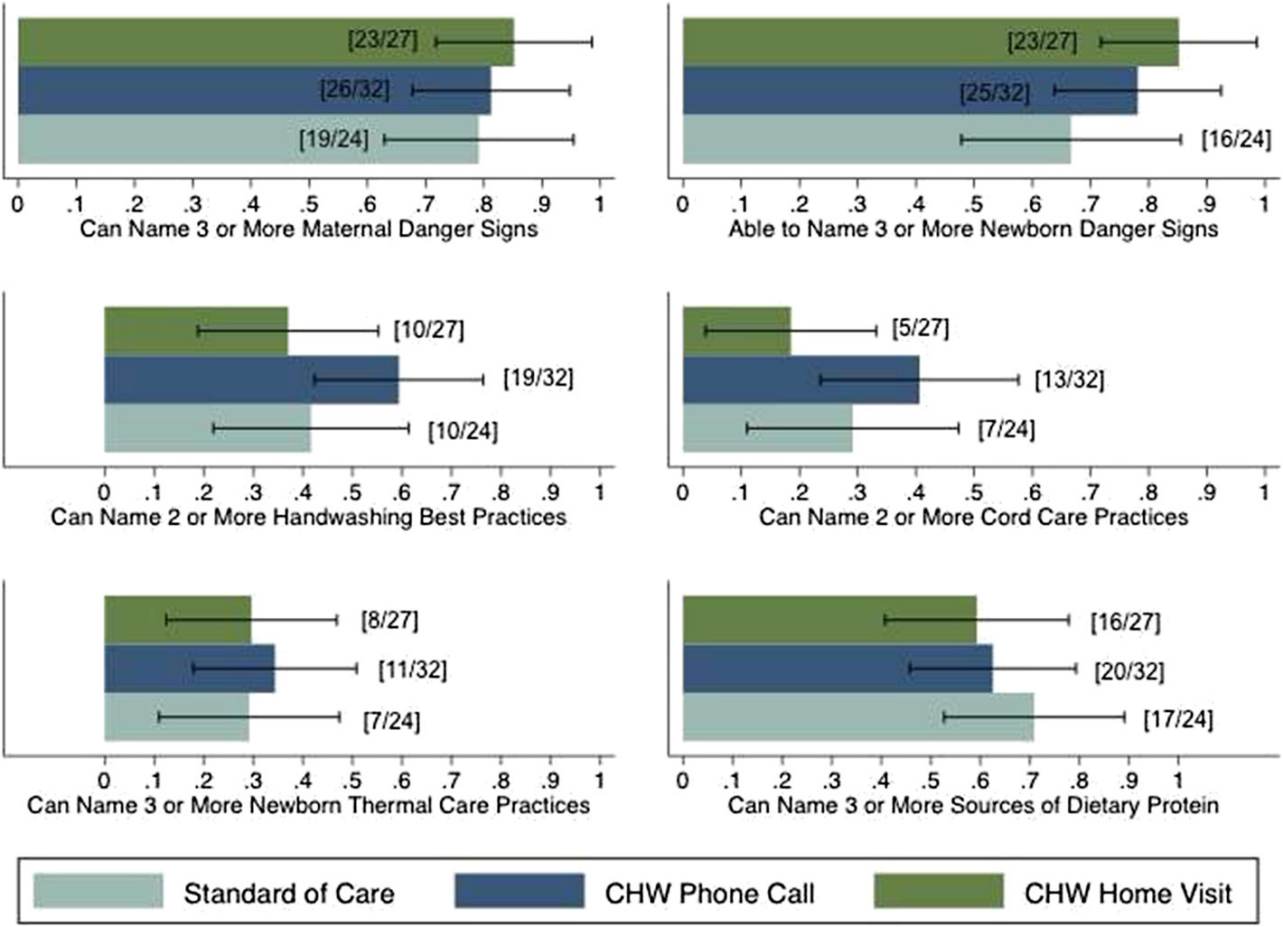
Self-reported knowledge of postnatal danger signs and health practices at day 10

Figure 2 presents the percentage of women in each treatment arm with knowledge of six key postnatal health knowledge items. 85 % (23/27) of women assigned the visit arm were able to name three or more maternal danger signs, as well as three or more infant danger signs. 81 % (26/32) of the phone call arm was able to name three or more maternal danger signs, with 78 % (25/32) able to name three or more infant danger signs. 79 % (19/24) and 67 % (16/24) of women in the standard of care arm able to name three or more maternal and infant danger signs, respectively.

### Health practices, reported at 10 days and 9 weeks postnatal

**Fig. 3:**
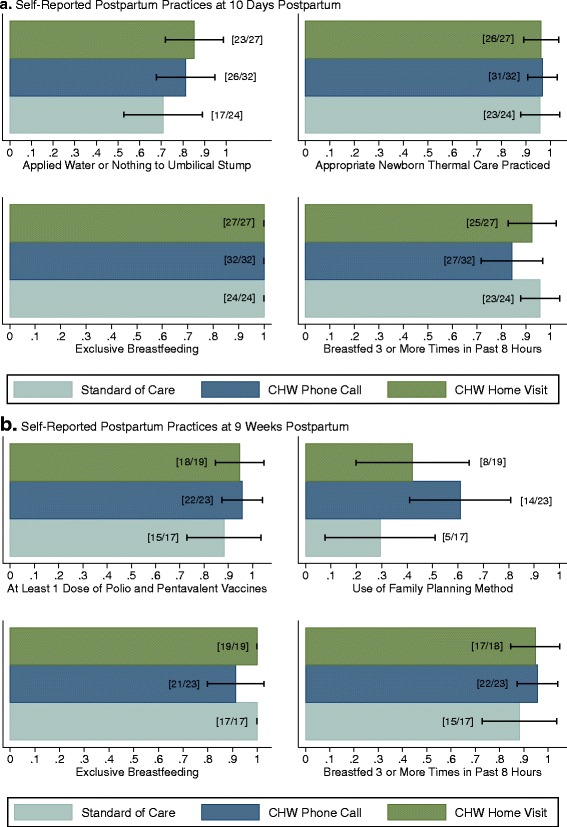
Self-reported postnatal health practices at 10 days and 9 weeks after delivery

Postnatal health practices at 10 days after delivery are presented in Panel A of Fig. 3. We find 100 % (83/83) of the surveyed sample reporting exclusive breastfeeding, 96 % (80/83) reporting practicing appropriate newborn thermal care practices while bathing, and 90 % (75/83) reporting frequent breastfeeding (defined as breastfeeding 3 or more times in the previous 8 hours). We find the highest level of variation in short-term postnatal health practices in umbilical cord care, with 85 % (23/27) of the home visit arm and 81 % (26/32) phone call arm reporting applying water or nothing to the stump (as recommended), compared to 71 % (17/24) of the standard of care arm. We find no statistically significant differences in short-term postnatal health practices by treatment assignment.

Self-reported health practices at nine weeks after delivery are presented in Panel B of Fig. 3. As with the short-term health practices, we find very high reported compliance with recommended health practices at nine weeks post-delivery. We find no significant differences in exclusive breastfeeding, frequency of breastfeeding, or receipt of routine infant immunizations by treatment arm. We find an increased likelihood of family planning use by nine weeks after delivery in both the phone call and home visit arms relative to the standard of care, with those assigned to the phone call having between 3.7 times (unadjusted 95 % CI: 0.97-14.4; *p* = 0.06.) and 3.1 times increased odds (adjusted 95 % CI: 0.51–18.7; *p* = 0.22) and those assigned to the home visit arm having between 1.7 times (unadjusted 95 % CI: 0.43-7.1; *p* = 0.43) and 2.2 times increased odds (adjusted 95 % CI: 0.35–13.4; *p* = 0.41) of reporting family planning, although these results are not statistically significant.^4^

In Table 6, we present regression estimates using ordinary least squares where the outcomes are summative indices of postnatal health knowledge at day 10 post-delivery (unadjusted model in column 1; adjusted model in column 2) and short- and long-term postnatal health practices assessed at 10 days and 9 weeks post-delivery, respectively (unadjusted model in column 3; adjusted model in column 4). We find an average knowledge in the standard of care arm of 3.2 items out of the maximum 6. While estimates of program impact of phone call assignment are positive in the unadjusted and adjusted models the differences from the standard of care group are not statistically significant.

**Table 6:** Self-reported postnatal health knowledge and practices

	(1)	(2)	(3)	(4)
	Index of postnatal health knowledge, as reported at day 10 post-delivery	Index of postnatal health knowledge, as reported at day 10 post-delivery	Index of postnatal health practices, as reported at day 10 and 9 weeks post-delivery	Index of postnatal health practices, as reported at day 10 and 9 weeks post-delivery
	*Unadjusted*	*Adjusted*	*Unadjusted*	*Adjusted*
*Estimation Type*	*OLS*	*OLS*	*OLS*	*OLS*
CHW Call	0.40	0.57	0.45	0.29
	(−0.48 - 1.28)	(−0.37 - 1.50)	(−0.18 - 1.08)	(−0.47 - 1.06)
CHW Home Visit	−0.019	0.11	0.58*	0.45
	(−0.82 - 0.78)	(−0.75 - 0.97)	(−0.012 - 1.17)	(−0.25 - 1.15)
Mean of standard of care arm	3.2	3.2	6.6	6.6
p-value for test of call = visit	0.323	0.331	0.649	0.650
p-value for test of joint significance of call and visit	0.564	0.457	0.148	0.430
Controls?	N	Y	N	Y
R-squared	0.016	0.045	0.073	0.158
Observations	83	79	52	49

*** *p* < 0.01, ** *p* < 0.05, * *p* < 0.1

Notes: Coefficients generated via OLS regression (models 1–4) are presented with 95 % confidence intervals constructed with robust standard errors. Postnatal health knowledge (models 1 and 2) is assessed as an ordinal variable generated using self-reported knowledge collected during the day 10 postnatal survey; knowledge is assessed as a summative index with a maximum of 6 points and a minimum of 0 points, where each point represents knowledge of the following 6 postnatal health topics: 1) ability to name 3 or more maternal danger signs; 2) ability to name 3 or more infant danger signs; 3) ability to name 2 or more hand washing best practices; 4) ability to name 2 or more; 5) ability to name 3 or more recognized newborn thermal care practices; 6) and ability to name 3 or more sources of maternal dietary protein. Postnatal health practices (models 3 and 4) assessed as an ordinal variable generated using self-reported practices collected during the day 10 and 9 week postnatal surveys; health behaviors are assessed as a summative index with a maximum of 8 points and a minimum of 0 points, where 4 points represent 4 key health practices reported at 10 day (exclusive breastfeeding, breastfed 3 or more times in the past 8 hours, appropriate newborn thermal care practices, and water or nothing applied to umbilical cord stump) and 4 points represent 4 key health practices reported at 9 weeks (exclusive breastfeeding, breastfed 3 or more times in the past 8 hours, use of postnatal contraception, infant has received at least one dose of polio and pentavalent vaccines). For all adjusted regressions (columns 2 and 4), individual level covariates include the female participant’s age (coded as an ordinal variable with values 18–25, 26–30, 31–35, or 36–40 years old), marital status (defined as a binary single or married), a binary variable indicating whether the female respondent was employed at any time in the past 12 months, a binary variable indicating whether the participant was enrolled in a concurrent randomized trial and received a voucher (cost subsidy) for postnatal family planning services, and a binary variable indicating whether the participant attended one or more antenatal care visits at a Jacaranda Health facility

Postnatal health practices at ten days and nine weeks are also assessed using a summative index. While estimates of a treatment effect are positive in both crude and adjusted models, they are substantively small and not statistically significant.

### Limitations

Our findings are subject to some important limitations. The sample size for this intervention was quite small. Our study was primarily powered to detect large effects of the intervention and thus our power to detect moderate effects was limited and most of the findings are not statistically significant at the 5 % level. Furthermore, our study was not designed to be powered to detect changes in health outcomes. A substantially larger trial would be needed to determine the effect of similar interventions on key health outcomes such as mortality.

Further, this intervention took place in an urban setting and within partnerships at a social enterprise providing private low-cost maternal and newborn health care. Further research is necessary to evaluate if the findings seen could be replicated within the public sector, which has a larger geographical scale but also faces other challenges. Because our intervention relies on facilities to identify women who have recently delivered, it would not be suitable for providing postnatal care to women who deliver at home. Therefore this implementation strategy for delivering postnatal care is likely most relevant for urban areas such as Nairobi where rates of home birth are low (11.6 %) [5].

Furthermore, because Jacaranda Health is a private maternity facility, the patient profile may not represent women who deliver at home or in public facilities. Comparing the socioeconomic indicators of women in our sample to characteristics of the national urban sample in Kenya we find that our sample is similar in terms of access to an improved water source, with access to an improved water source for 90.8 % of urban households in Kenya and 93 % (71/76) of households in our sample [4]. Our sample has higher rates of access to improved non-shared toilet facilities at (41 %) compared to the national urban average of 30 % [4].

Postnatal knowledge outcomes indicate that health knowledge was uniformly and unexpectedly high in our sample. In the context of relatively high overall knowledge, our estimate of the impact of the checklist interventions do not point to impacts of the intervention on knowledge. Replication of this evaluation may elucidate the potential knowledge gains of such an intervention in a less informed sample. Finally, while we were able to observe health seeking behavior through administrative data shared by our partner, many of the health practices measured in the study (such as breastfeeding and cord care practices) are self-reported. Some of the high reported rates of beneficial practices such as dry cord care and exclusive breastfeeding may be due to reporting bias.

Our study also had significant attrition, both in the implementation of the interventions and tracking of respondents at the ten-day follow-up survey and nine week follow-up survey. Estimates presented in Additional files 2, 3, 4, 5, 6 focus on a subsample that could be reached for the day 3 interventions. While the checklist delivered over the mobile phone was able to reach a larger share of respondents than the checklist administered in person, substantive conclusions are largely similar in both samples.

## Discussion

Our findings suggest that CHWs can deliver an algorithmic, checklist-based postnatal health intervention either by phone or in a visit to women’s homes three days after they give birth. Overall, the checklist was administered for 68 % (50/73) of women who gave birth within three days of their delivery with more women reached over the phone than in person. Our study provides novel evidence of the feasibility of using checklists to support the work of CHWs in administering postnatal care in person and over mobile phones. While previous interventions to improve outcomes for mother and baby during the postpartum period have been complex and multifaceted, potentially involving as many as 11 visits over the entire period of a pregnancy [7, 8, 13, 24, 25], our intervention focused narrowly on postnatal care, piloting a mobile phone based version of a postnatal checklist that can be administered across a variety of health systems under different resource constraints. One advantage of this simple stand-alone approach is it addresses the concern that the wide scope of comprehensive home visit interventions presents a challenge to consistent distribution or intervention coverage to women and their newborns within existing community delivery systems [25].

Our intervention innovates by comparing the effectiveness of delivering a postnatal checklist entirely in person during a home visit or over mobile phones. Tools that leverage mHealth technology pose an opportunity to supplement existing community delivery systems, like CHW home visits, by increasing accessibility and coverage [26–28]. CHW home visit interventions in South Africa used mobile phone technology for data collection, patient registry, and health worker performance management, but not as the main point of contact for the intervention [8]. In urban areas, such as the ones we focus on in our study, mobile phone use is widespread and represents an opportunity for delivery of interventions that reach women in their homes.

Our study provides specific evidence on how postnatal checks can influence women’s likelihood of seeking care and lead to earlier care-seeking in the crucial window immediately after birth. Overall, while some of our impact estimates are imprecise, the direction and magnitude of our findings suggest that the home visit administration of the checklist was especially helpful in encouraging timely care-seeking in the critical postnatal window. The home visit increases the salience of infant health complications or concerns, with women assigned to the home visit treatment arm significantly more likely to report infant health problems at ten days after giving birth compared to the standard of care arm and more likely to both recognize and take action on complications detected in their newborns.

The evidence that providing assistance and support to women in this window of time, which is both critical for their health and their newborns health but also filled with many other responsibilities and stresses, can increase timely care-seeking is consistent with evidence from Warren et al. (2015) that vouchers providing reimbursement for reproductive health services in Kenya can increase the number of newborns seen by facilities within their first 48 hours [29]. The significance of reducing delays in care-seeking is particularly important in the context of the peri-urban poor, where delays in care-seeking are common [30]. Such earlier linkages to facility-based care could have health implications, particularly as the first week of life poses significant risks. These risks are particularly important because the rapid progression of many neonatal illnesses, such as sepsis, pose notable risks to neonatal survival [31].

Our results highlight the limited care seeking behavior in the postpartum period for maternal health specifically. While newborns were brought to a health facility for care within 10 days of delivery 96 % of the time in the standard of care arm (in 23 out of 24 cases), mothers receive care in a facility only 8 % of the time (in 2 out of 24 cases). We see suggestive evidence that our interventions increased the likelihood of reporting a maternal complication and reduced delays in seeking care for maternal complications compared to the standard of care, though these differences are not statistically significant at conventional levels. Overall knowledge of both maternal and newborn danger signs is high across both standard of care and intervention arms in our sample, which suggests that addressing complications in both the immediate postnatal period and beyond would require increasing the likelihood of acting on perceived maternal health complications.

Across all three arms, we see high levels of knowledge and rates of beneficial postnatal practices such as breastfeeding, cord-care and vaccinations in our sample, resulting in very few statistically significant differences across the intervention treatment arms. We also see few substantive or statistically significant differences in practices and knowledge outcomes at the longer-term follow-up at 9 weeks across treatment arms, though we do see some suggestive but not statistically significant evidence of increases in the likelihood of using modern contraceptives. This evidence is consistent with findings from Watt et al. (2015) that one of the primary ways that the Safe Motherhood Voucher program in Kenya improved postnatal care was to improve counseling about family planning [32].

## Conclusions

We find that a simple checklist, administered by CHWs over mobile phones or in person three days after giving birth, leads to earlier utilization of postnatal care and better recognition of potential mother and baby complications. Our evidence innovates by comparing delivery models for a targeted postnatal intervention that could be widely and affordably scaled.

## Abbreviation

CHW, Community Health Worker.

## Additional files

Additional file 1: Postpartum screening checklist. (DOCX 1253 kb) Additional file 2: Postpartum care-seeking behaviors, health problems and responses, and postpartum knowledge and health behaviors, among subsample of participants reached by the day 3 postpartum interventions. (DOCX 110 kb) Additional file 3: Participant flow for subsample of participants reached by the day 3 postpartum interventions. (JPG 207 kb) Additional file 4: Complications detected, referred, and referrals acted upon, among subsample of participants reached by the day 3 postpartum interventions. (JPG 45 kb) Additional file 5: Self-reported knowledge of postnatal danger signs and health practices at day 10, among subsample of participants reached by the day 3 postpartum interventions. (JPG 80 kb) Additional file 6: Self-reported postnatal health practices at 10 days and 9 weeks after delivery, among subsample of participants reached by the day 3 postpartum interventions. (PDF 61 kb)

## Footnotes

1 In order to avoid the possibility that patients responded to the phone call with more health seeking behaviors because of reciprocity, we include a phone call for the standard of care group that focused exclusively on customer service. Patients reported appreciating receiving any phone call so, to the extent that this call may have influenced behavior in the standard of care group, some estimates of program impacts may be underestimates.

2 While our assessment guidelines are consistent with the WHO guidelines from 2014, our postnatal checklist was developed in 2013, prior to the release of these guidelines and was therefore not specifically based on these guidelines.

3 Referrals were made to the nearest facility (not to Jacaranda) since Jacaranda was not offering emergency comprehensive pediatric care at the time of the intervention.

4 We find highest uptake of the hormonal implant in both the standard of care and home visit arms (comprising 60% (3/5) and 50 % (4/8) of participants reporting contraceptive use assigned to these arms, respectively). In the phone-administered checklist arm, the most common method was oral contraceptives (comprising 50 % of participants in this arm). In both the phone- and home visit-administered checklist arms, injectables were the second most common method (comprising 29 % (4/14) and 25 % (2/8) of participants reporting contraceptive use in these arms, respectively).
