# Supplementary material for: Can a community health worker administered postnatal checklist increase health-seeking behaviors and knowledge?: evidence from a randomized trial with a private maternity facility in Kiambu County, Kenya
Source: BMC Pregnancy Childbirth. 2016 Jun 4;16:136. doi: 10.1186/s12884-016-0914-z (PMC4893209; doi:10.1186/s12884-016-0914-z)
Supplement: Additional file 2: — Postpartum care-seeking behaviors, health problems and responses, and postpartum knowledge and health behaviors, among subsample of participants reached by the day 3 postpartum interventions. (DOCX 110 kb) [file 12884_2016_914_MOESM2_ESM.docx]

# Table A1. Postpartum care-seeking behaviors for mother and newborn as reported during day 10 survey, among subgroup of participants reached by the Day 3 postpartum intervention

| **Panel A. Maternal Postpartum Care Seeking** | | | | |
| --- | --- | --- | --- | --- |
|  | (1) | (2) | (3) | (4) |
|  | Any maternal postpartum care sought | Any maternal postpartum care sought | Days postpartum maternal care sought, among maternal care-seekers | Days postpartum maternal care sought, among maternal care-seekers^‡^ |
|  | *Unadjusted* | *Adjusted* | *Unadjusted* | *Adjusted* |
| *Estimation type* | *Logistic* | *Logistic* | *OLS* | *OLS* |
|  |  |  |  |  |
| CHW phone call | 2.10 | 2.70 | -1 | 0.40 |
|  | (0.34 - 12.9) | (0.37 - 19.5) | (-4.80 - 2.80) | (-3.83 - 4.63) |
| CHW home visit | 3.00 | 11.5** | -4.25*** | -4.20 |
|  | (0.48 - 18.9) | (1.05 - 125) | (-6.96 - -1.54) | (-10.6 - 2.15) |
| Control arm mean | 0.09 | 0.09 | 8.5 | 8.5 |
|  |  |  |  |  |
| p-value for test of call = visit | 0.654 | 0.106 | 0.128 | 0.089 |
|  |  |  |  |  |
| p-value for test of joint significance of call and visit | 0.503 | 0.115 | 0.022 | 0.164 |
|  |  |  |  |  |
| Controls? | N | Y | N | Y |
|  |  |  |  |  |
| R-squared |  |  | 0.444 | 0.963 |
|  |  |  |  |  |
| Observations | 65 | 62 | 10 | 9 |
|  |  |  |  |  |
| **Panel B. Newborn Postpartum Care Seeking** | | | | |
|  | (1) | (2) | (3) | (4) |
|  | Any newborn postpartum care sought^‡‡^ | Any newborn postpartum care sought^‡^ | Days postpartum newborn care sought, among newborn care-seekers | Days postpartum newborn care sought, among newborn care-seekers |
|  | *Unadjusted* | *Adjusted* | *Unadjusted* | *Adjusted* |
| *Estimation type* | *Logistic* |  | *OLS* | *OLS* |
|  |  |  |  |  |
| CHW phone call | 1.05 | 0.67 | -2.04** | -2.21** |
|  | (0.060 - 18.2) | (0.069 - 6.47) | (-3.78 - -0.31) | (-3.95 - -0.46) |
| CHW home visit | 0.77 | 0.33 | -2.29*** | -2.23** |
|  | (0.044 - 13.6) | (0.017 - 6.28) | (-4.00 - -0.59) | (-4.12 - -0.34) |
| Mean of comparison group | 0.95 | 0.95 | 6 | 6 |
|  |  |  |  |  |
| p-value for test of call = visit | 0.836 | 0.616 | 0.725 | 0.980 |
|  |  |  |  |  |
| p-value for test of joint significance of call and visit | 0.975 | 0.759 | 0.025 | 0.032 |
|  |  |  |  |  |
| Controls? | N | Y | N | Y |
|  |  |  |  |  |
| R-squared |  |  | 0.136 | 0.238 |
|  |  |  |  |  |
| Observations | 65 | 32 | 62 | 59 |
|  |  |  |  |  |

*** p<0.01, ** p<0.05, * p<0.1

^‡^ The binary covariate for receipt of a postnatal contraception subsidy is dropped due to collinearity.

^‡‡^ The reduced number of observations in Panel B (1) is due to the low variation in the outcomes across treatment arms, with 96.6% of the total sample reporting having sought newborn care by the day 10 survey. In model (1), the coefficient on the CHW phone call intervention cannot be estimated due to this lack of variation in the outcome relative to the control arm. Likewise, no adjusted model is presented in column (2) due to the reduced number of observations when covariates are included in the model.

Notes: Odds ratios generated via logistic regression (models 1, 2) are presented with 95% confidence intervals constructed with robust standard errors. Maternal care seeking (panel A, columns 1 and 2) is defined as a binary variable, taking on the value of 1 if the woman reports seeking any maternal-related care (including postnatal care, breastfeeding support, or care-seeking for maternal health problems). Infant care seeking (panel B, columns 1 and 2) is defined as a binary variable, taking on the value of 1 if the woman reports seeking any maternal-related care (including postnatal care, breastfeeding support, or care-seeking for maternal health problems). OLS coefficients are presented for models 3 and 4. Days postpartum maternal care sought (panel A, columns 3 and 4) is defined as a continuous variable of the number of days that maternal care was sought after the date of delivery. Days postpartum infant care sought (panel B, columns 3 and 4) is likewise defined as a continuous variable of the number of days that care for the infant was sought after the date of delivery. For all adjusted regressions (columns 2 and 4), individual level covariates include the female participant’s age (coded as an ordinal variable with values 18-25, 26-30, 31-35, or 36-40 years old), marital status (defined as a binary single or married), a binary variable indicating whether the female respondent was employed at any time in the past 12 months, a binary variable indicating whether the participant was enrolled in a concurrent randomized trial and received a voucher (cost subsidy) for postnatal family planning services, and a binary variable indicating whether the participant attended one or more antenatal care visits at a Jacaranda Health facility.

# Table A2. Postpartum health problems and responses as reported during day 10 survey, among subgroup of participants reached by the Day 3 postpartum intervention

| Panel A. Maternal health problem reporting and responses | | | | |
| --- | --- | --- | --- | --- |
|  | (1) | (2) | (3) | (4) |
|  | Any maternal health problem reported | Any maternal health problem reported | Maternal problem reported and action taken | Maternal problem reported and action taken |
|  | *Unadjusted* | *Adjusted* | *Unadjusted* | *Adjusted* |
| *Estimation type* | *Logistic* | *Logistic* | *Logistic* | *Logistic* |
|  |  |  |  |  |
| CHW Call | 0.61 | 0.34 | 0.95 | 0.68 |
|  | (0.090 - 4.07) | (0.029 - 3.99) | (0.12 - 7.53) | (0.054 - 8.64) |
| CHW Home Visit | 3.33 | 8.43* | 1.31 | 2.41 |
|  | (0.69 - 16.1) | (0.91 - 78.2) | (0.16 - 10.5) | (0.25 - 23.1) |
| Mean of comparison group | 0.13 | 0.13 | 0.09 | 0.09 |
|  |  |  |  |  |
| p-value for test of call = visit | 0.058 | 0.006 | 0.764 | 0.363 |
|  |  |  |  |  |
| p-value for test of joint significance of call and visit | 0.111 | 0.016 | 0.949 | 0.619 |
|  |  |  |  |  |
| Controls? | N | Y | N | Y |
|  |  |  |  |  |
| Observations | 65 | 62 | 65 | 42 |
| Panel B. Newborn health problem reporting and responses | | | | |
|  | (1) | (2) | (3) | (4) |
|  | Any newborn health problem reported | Any newborn health problem reported | Newborn problem reported and action taken | Newborn problem reported and action taken |
|  | *Unadjusted* | *Adjusted* | *Unadjusted* | *Adjusted* |
| *Estimation type* | *Logistic* | *Logistic* | *Logistic* | *Logistic* |
|  |  |  |  |  |
| CHW Call | 1.48 | 1.44 | 1.33 | 1.03 |
|  | (0.39 - 5.64) | (0.36 - 5.79) | (0.26 - 6.82) | (0.19 - 5.53) |
| CHW Home Visit | 4.50** | 4.37* | 3.33 | 3.46 |
|  | (1.14 - 17.7) | (0.97 - 19.7) | (0.69 - 16.1) | (0.66 - 18.1) |
| Mean of comparison group | 0.22 | 0.22 | 0.13 | 0.13 |
|  |  |  |  |  |
| p-value for test of call = visit | 0.092 | 0.143 | 0.220 | 0.150 |
|  |  |  |  |  |
| p-value for test of joint significance of call and visit | 0.077 | 0.144 | 0.262 | 0.240 |
|  |  |  |  |  |
| Controls? | N | Y | N | Y |
|  |  |  |  |  |
| Observations | 65 | 62 | 65 | 62 |

*** p<0.01, ** p<0.05, * p<0.1

Notes: Odds ratios generated via logistic regression (models 1-4) are presented with 95% confidence intervals constructed with robust standard errors. “Any maternal problem reported” is a binary variable indicating whether or not the participant reported any maternal-related health problems at 10 days postpartum. “Maternal problem reported and action taken” is a binary variable taking the value 1 if the respondent reported both any maternal health problem or concern and taking any action to address the problem, including calling a health facility or pharmacy, visiting a facility or pharmacy, or another action. “Any infant problem reported” is a binary variable indicating whether or not the participant reported any infant-related health problems at 10 days postpartum. “Newborn problem reported and action taken” is a binary variable taking the value 1 if the respondent reported both any newborn health problem or concern and taking any action to address the problem, including calling a health facility or pharmacy, visiting a facility or pharmacy, or another action. For all adjusted regressions (columns 2 and 4), individual level covariates include the female participant’s age (coded as an ordinal variable with values 18-25, 26-30, 31-35, or 36-40 years old), marital status (defined as a binary single or married), a binary variable indicating whether the female respondent was employed at any time in the past 12 months, a binary variable indicating whether the participant was enrolled in a concurrent randomized trial and received a voucher (cost subsidy) for postnatal family planning services, and a binary variable indicating whether the participant attended one or more antenatal care visits at a Jacaranda Health facility.

# Table A3. Self-reported postpartum knowledge and health behaviors, among subgroup of participants reached by the Day 3 postpartum intervention

|  | (1) | (2) | (3) | (4) |
| --- | --- | --- | --- | --- |
|  | Index of postpartum health knowledge, as reported at day 10 postpartum | Index of postpartum health knowledge, as reported at day 10 postpartum | Index of postpartum health behaviors, as reported at day 10 and 9 weeks postpartum | Index of postpartum health behaviors, as reported at day 10 and 9 weeks postpartum |
|  | *Unadjusted* | *Adjusted* | *Unadjusted* | *Adjusted* |
| *Estimation type* | *OLS* | *OLS* | *OLS* | *OLS* |
|  |  |  |  |  |
| CHW Call | 0.33 | 0.37 | 0.56 | 0.27 |
|  | (-0.66 - 1.32) | (-0.74 - 1.47) | (-0.15 - 1.26) | (-0.59 - 1.14) |
| CHW Home Visit | 0.19 | 0.35 | 0.45 | 0.30 |
|  | (-0.70 - 1.08) | (-0.57 - 1.27) | (-0.21 - 1.11) | (-0.43 - 1.03) |
| Mean of comparison group | 3.1 | 3.1 | 6.6 | 6.6 |
|  |  |  |  |  |
| p-value for test of call = visit | 0.784 | 0.976 | 0.736 | 0.943 |
|  |  |  |  |  |
| p-value for test of joint significance of call and visit | 0.793 | 0.684 | 0.245 | 0.676 |
|  |  |  |  |  |
| Controls? | N | Y | N | Y |
|  |  |  |  |  |
| R-squared | 0.008 | 0.061 | 0.079 | 0.199 |
|  |  |  |  |  |
| Observations | 62 | 62 | 40 | 38 |

*** p<0.01, ** p<0.05, * p<0.1

Notes: Coefficients generated via OLS regression (models 1-4) are presented with 95% confidence intervals constructed with robust standard errors. Postpartum health knowledge (models 1 and 2) is assessed as an ordinal variable generated using self-reported knowledge collected during the day 10 postpartum survey; knowledge is assessed as a summative index with a maximum of 6 points and a minimum of 0 points, where each point represents knowledge of the following 6 postpartum health topics: 1) ability to name 3 or more maternal danger signs; 2) ability to name 3 or more infant danger signs; 3) ability to name 2 or more hand washing best practices; 4) ability to name 2 or more; 5) ability to name 3 or more recognized newborn thermal care practices; 6) and ability to name 3 or more sources of maternal dietary protein. Postpartum health behaviors (models 3 and 4) assessed as an ordinal variable generated using self-reported behaviors collected during the day 10 and 9 week postpartum surveys; health behaviors are assessed as a summative index with a maximum of 8 points and a minimum of 0 points, where 4 points represent 4 key health behaviors reported at 10 day (exclusive breastfeeding, breastfed 3 or more times in the past 8 hours, appropriate newborn thermal care practices, and water or nothing applied to umbilical cord stump) and 4 points represent 4 key health behaviors reported at 9 weeks (exclusive breastfeeding, breastfed 3 or more times in the past 8 hours, use of postpartum contraception, infant has received at least one dose of polio and pentavalent vaccines). For all adjusted regressions (columns 2 and 4), individual level covariates include the female participant’s age (coded as an ordinal variable with values 18-25, 26-30, 31-35, or 36-40 years old), marital status (defined as a binary single or married), a binary variable indicating whether the female respondent was employed at any time in the past 12 months, a binary variable indicating whether the participant was enrolled in a concurrent randomized trial and received a voucher (cost subsidy) for postnatal family planning services, and a binary variable indicating whether the participant attended one or more antenatal care visits at a Jacaranda Health facility.
