# Supplementary figures and images for: Can a community health worker administered postnatal checklist increase health-seeking behaviors and knowledge?: evidence from a randomized trial with a private maternity facility in Kiambu County, Kenya
Source: BMC Pregnancy Childbirth. 2016 Jun 4;16:136. doi: 10.1186/s12884-016-0914-z (PMC4893209; doi:10.1186/s12884-016-0914-z)

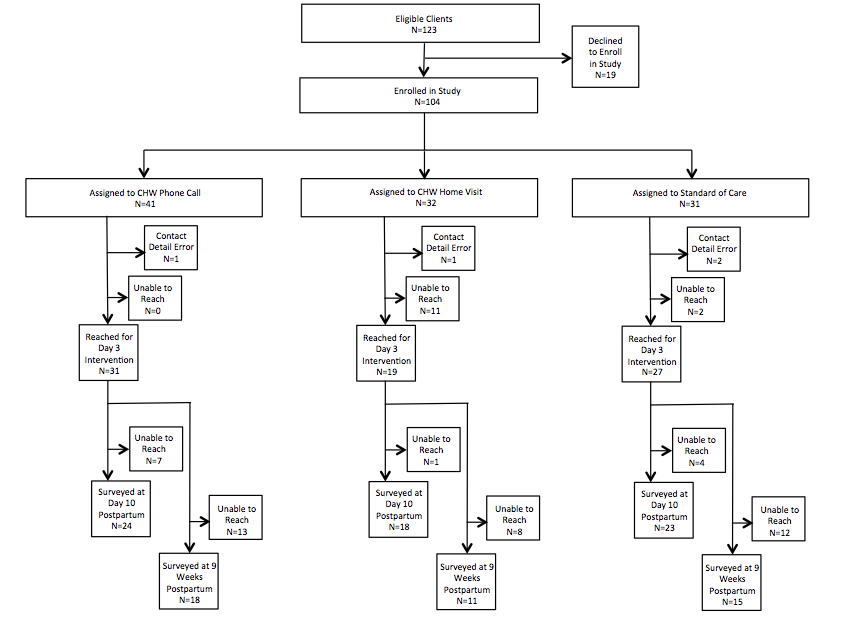

Supplement: Additional file 3: — Participant flow for subsample of participants reached by the day 3 postpartum interventions. (JPG 207 kb) [file 12884_2016_914_MOESM3_ESM.jpg]

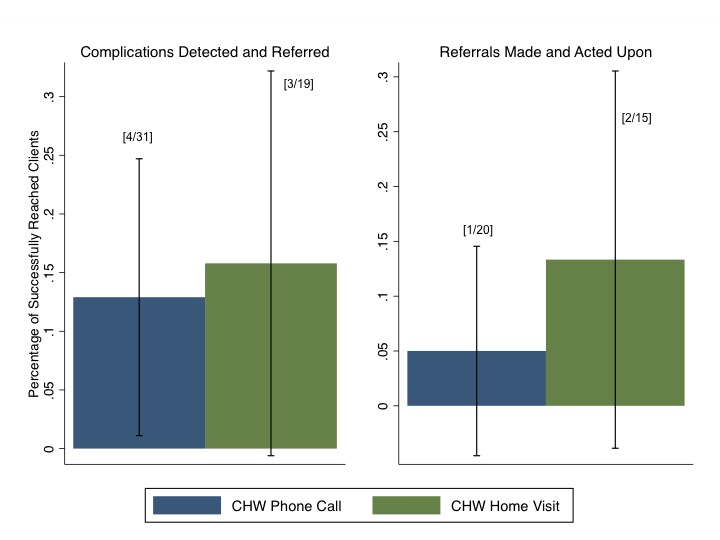

Supplement: Additional file 4: — Complications detected, referred, and referrals acted upon, among subsample of participants reached by the day 3 postpartum interventions. (JPG 45 kb) [file 12884_2016_914_MOESM4_ESM.jpg]

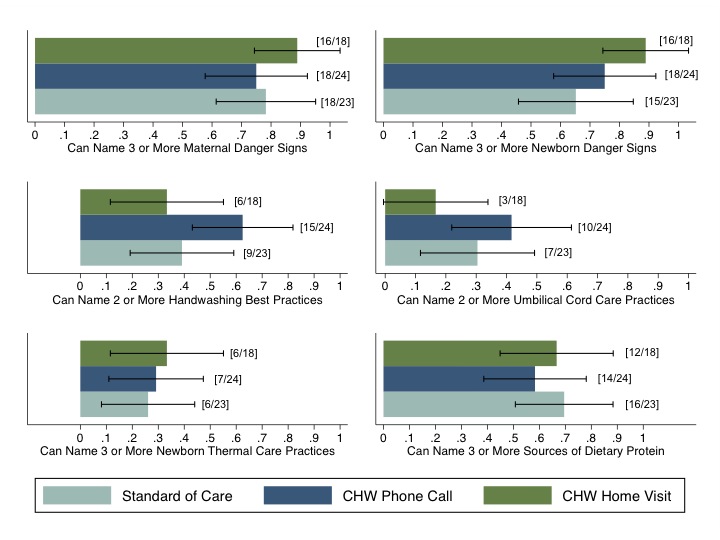

Supplement: Additional file 5: — Self-reported knowledge of postnatal danger signs and health practices at day 10, among subsample of participants reached by the day 3 postpartum interventions. (JPG 80 kb) [file 12884_2016_914_MOESM5_ESM.jpg]
