# Supplementary material for: Can a community health worker administered postnatal checklist increase health-seeking behaviors and knowledge?: evidence from a randomized trial with a private maternity facility in Kiambu County, Kenya
Source: BMC Pregnancy Childbirth. 2016 Jun 4;16:136. doi: 10.1186/s12884-016-0914-z (PMC4893209; doi:10.1186/s12884-016-0914-z)

Panel A. Self-Reported Postpartum Behaviors at 10 Days Postpartum

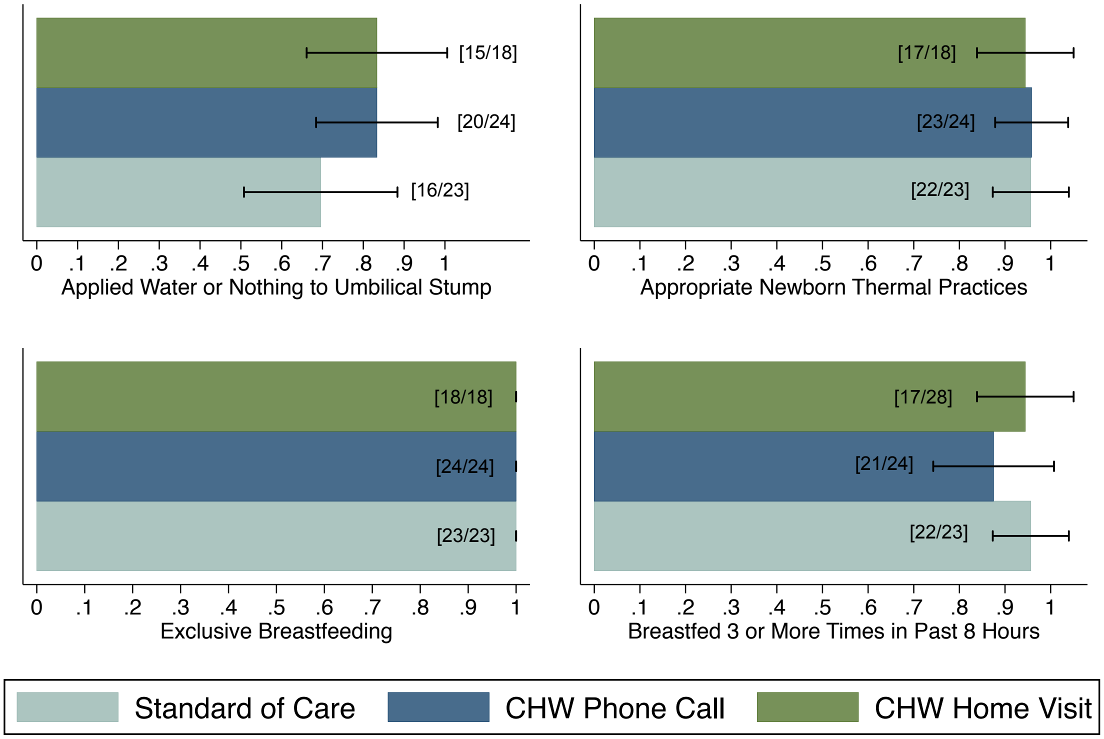

Panel B. Self-Reported Postpartum Behaviors at 9 Weeks Postpartum

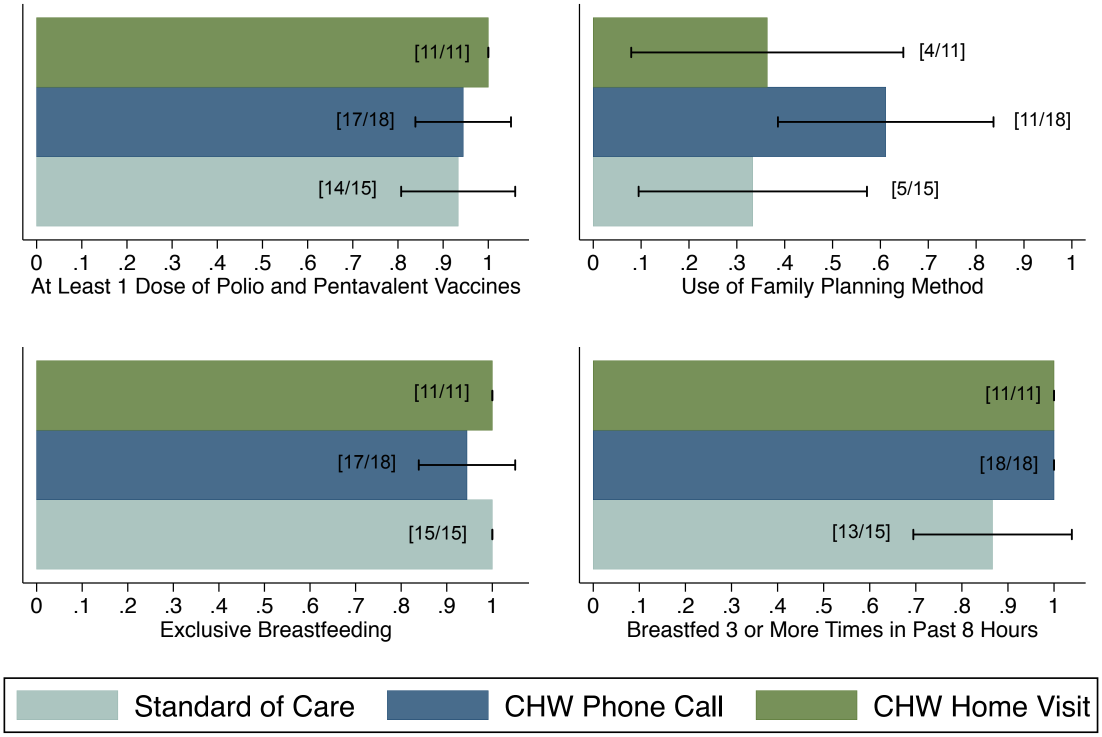

Supplement: Additional file 6: — Self-reported postnatal health practices at 10 days and 9 weeks after delivery, among subsample of participants reached by the day 3 postpartum interventions. (PDF 61 kb) [file 12884_2016_914_MOESM6_ESM.pdf]
