# Supplementary material for: Can a community health worker administered postnatal checklist increase health-seeking behaviors and knowledge?: evidence from a randomized trial with a private maternity facility in Kiambu County, Kenya
Source: BMC Pregnancy Childbirth. 2016 Jun 4;16:136. doi: 10.1186/s12884-016-0914-z (PMC4893209; doi:10.1186/s12884-016-0914-z)
Supplement: Supplementary file 4 [file 12884_2016_914_MOESM1_ESM.docx]

JACARANDA MATERNITY **Postpartum Home Follow-Up Program**

POSTPARTUM SCREENING CHECKLIST

Complete for each call or home visit postpartum.

PROVIDER ID__________DATE_________________TIME STARTED____________ TIME ENDED________________

**Patient Information**			
**Name:**	**Contact Number:**<br>**Alternate contact number:**		
**Jacaranda ID**	**Parity**		
**Actual Delivery Date**	**Date of Discharge**	**Postpartum Day** (number)	
**Mother's Screening**		**Abnormal**	**Normal**
First, I am going to ask some questions about how you are feeling since you left Jacaranda Maternity…			
Since you came home from Jacaranda Maternity, have you had fever or chills?		☐ Yes	☐ No
It is normal for women to experience vaginal discharge, but some discharge is a sign that you should see a nurse. Since your delivery, have you noticed any vaginal discharge that had a bad odor?		☐ Yes	☐ No
Since your delivery, have you experienced convulsions or loss of consciousness?		☐ Yes	☐ No
Since you came home from Jacaranda Maternity, have you had bleeding where you are soaking through a pad every hour?		☐ Yes	☐ No
Since your delivery, have you experienced any headaches, dizziness, or faintness?		☐ Yes	☐ No
Since your delivery, have you had any blurry vision or unusual trouble seeing?		☐ Yes	☐ No
Have you had any increased cramping or abdominal pain?		☐ Yes	☐ No
Have you had increased perineal pain (the area between your vagina and anus) that did not get better with pain medicine?		☐ Yes	☐ No
Since your delivery, have you experienced any difficulty breathing?		☐ Yes	☐ No
Have you had any difficulty passing urine?		☐ Yes	☐ No
Most women find that their breasts are painful and swollen after delivery. Have you noticed any redness in your breasts or tenderness that is hot to the touch?		☐ Yes	☐ No
Have you noticed that your nipples are cracked and painful?		☐ Yes	☐ No
Have you noticed any lumps in your breasts or swelling of your lymph nodes in your under-arms?		☐ Yes	☐ No
Please find a mirror, and pull your bottom eyelid down slightly. Does the red part look lighter than normal?		☐ Yes	☐ No
"Please look at the palms of your hands, do those look lighter than normal?		☐ Yes	☐ No
**Baby's Screening**		**Abnormal**	**Normal**
Next, I am going to ask some questions about how your baby is doing since you left Jacaranda Maternity…			
How many times has your baby breastfed in the past 8 hours?		☐ 0-2 times	☐ 3 or more
Look at the whites of your baby's eyes. Do they appear yellow in colour?		☐ Yes	☐ No
Look at your baby's eyes, again. Do they appear red, swollen, or do you see any discharge draining?		☐ Yes	☐ No
Press gently on your baby's palms and soles of your baby's feet. As the colour returns to does it appear yellow?		☐ Yes	☐ No
Is your baby sleepy all the time or not moving much?		☐ Yes	☐ No

Is your baby not responsive to being touched or crying less?	☐ Yes	☐ No
Listen carefully to your baby's breathing, is your baby making grunting noises when breathing?	☐ Yes	☐ No
Look at your baby's nose, do you see his or her nostrils flaring or widening as he or she breathes in?	☐ Yes	☐ No
It is very important to keep your baby warm. Your baby's skin should be the same as the temperature of a normal adult. When you touch your baby's skin, do you notice that it feels colder than normal?	☐ Yes	☐ No
Touch your baby's belly, does he or she feel hot in comparison to your body?	☐ Yes	☐ No
Has your baby had any fits? For example, have you noticed your baby suddenly become very stiff or started to shake?	☐ Yes	☐ No
Look at your baby's skin, do you see red spots or blisters that contain pus on different body parts?	☐ Yes	☐ No
Look at the areas on your baby's belly where the cord was attached. Is it red, swollen, or draining pus?	☐ Yes	☐ No

Counseling

✓ Mark if completed

☐ **Breastfeeding**
- ☐Feeding every 2-3 hours (3 or more times in 8 hrs, 8 per day)
- ☐Feed at night and day
- ☐Signs of good attachment: CALM
  - ☐C-baby's Chin touching breast
  - ☐A-more Areola (dark part of nipple) seen above than below the baby's mouth,
  - ☐L-Lower Lip turned outwards and
  - ☐M-baby's Mouth open wide

☐ **Danger Signs: Mother**
- ☐Severe bleeding (more than 1 pad in an hr)
- ☐Severe lower abdominal pain
- ☐ Foul smelling vaginal discharge
- ☐Fever
- ☐Severe pain/swelling redness of perineum
- ☐Difficulty urinating
- ☐Headaches/blurry vision
- ☐Heat, pain or lump in breast

☐ **Danger Signs: Newborn**
- ☐Fever
- ☐Jaundice (yellow palms, eyes, soles)
- ☐Not feeding well
- ☐Lethargic/Not responsive to touch
- ☐Redness, discharge, foul smell from umbilical cord
- ☐Convulsions/fits
- ☐Abnormal breathing (grunting, nasal flaring, fast breathing)
- ☐Redness or pus from eyes

☐ **Cord care**
- ☐Keep cord clean and dry ☐ No applying anything to stump ☐Stump outside nappy

☐ **Thermal care**
- ☐Dress newborn in several layers of clothes ☐Bathe baby quickly in warm water, dry and dress quickly
- ☐ Warm room where mother and baby stay

Postpartum Home Follow-Up Program

- ☐ **Hand hygiene**
  ☐After using the toilet ☐Before touching or holding baby ☐ After washing or disposing of newborn nappies

☐**Family planning**

☐Healthy spacing 2 years ☐Can get pregnant again after 6 wks--partially breastfeeding, 6 months if exclusively breastfeeding

☐Ask for FP education or decision during ANC/Delivery Discharge

____

☐Ask which methods used in the past (remove methods that did not work)

☐Ask about desire for future children (if yes, then remove vasectomy and tubal ligation)

☐Ask about plans for breastfeeding (if yes, remove COCs and Monthly injectables. if remove LAM)

☐Ask if partner can use condoms(if no support, remove condoms)

☐Ask if they can avoid sex on certain unsafe days (if no, then remove Two Day Method and SDM)

☐ Place all cards in order of effectiveness

☐IUCDs can be used for up to 12 yrs ☐ IUCDs cannot move anywhere else in the body ☐IUCDs cannot cause infertility ☐Implants can be used for 3-5 yrs ☐Implants cannot move anywhere else in the body ☐Implants cannot cause infertility ☐ To make breastfeeding a birth spacing method a woman has to breastfeed exclusively (every 2-3 hours, night and day, not give baby supplemental foods, and no return of periods)

☐**Family Planning Method Decision from Balanced Counseling**

☐None ☐Injectables ☐POPs ☐IUCD ☐Implants

- ☐ **Increasing comfort postpartum**
  ☐Sitz baths ☐Walking ☐Pain medication (paracetamol/brufen)

- ☐ **Maternal nutrition**
  ☐Take additional fluids and food ☐Important to take protein (types include beans, meat, lentils, fish, chicken)

Comments and Jacaranda Follow-Up

Document any referrals, concerns, or questions for Jacaranda Maternity follow up

CHW Name:_________________________________ **CHW Signature:**_________________________________

Date:__________
